# Cerebral Symptoms and Hæmoptysis in Pneumonia

**Published:** 1906-06-23

**Authors:** 


### CEREBRAL SYMPTOMS AND HÆMOPTYSIS IN PNEUMONIA.

In the course of a lecture upon some of the clinical aspects of pneumonia Dr. Donald Hood [1] deals at length with two important and misleading varieties of this disease. The first is that variety which simulates intra-cranial mischief, the cerebral type of pneumonia. This type is most often associated with a lesion of the apex, and occurs most frequently in young subjects. The cerebral symptoms are paramount, and may include headache, intolerance of light, vomiting, delirium, fits of screaming, retraction of the head, and restlessness. This type has long been recognised. Henoch speaks of it as follows: "The latency of the physical symptoms, which may last from four to six days, along with the prominence of the cerebral and gastric symptoms, readily leads to the mistaken diagnosis of meningitis, or of typhoid fever, or even of intermittent fever, as I experienced in one case. Perhaps in such cases the pneumonia spreads gradually from the centre to the periphery, and only when it has reached this situation do the signs of consolidation appear distinctly. When this does take place the gastric or cerebral symptoms which have hitherto been prominent become less so, and the diagnosis at once becomes clear, but in some cases not until the fever is distinctly on the decline, or may even have ended critically."

These cases are grouped by Dr. Hood as follows: (1) Those in which the cerebral symptoms precede consolidation by some days; (2) those in which the physical signs of pneumonia are concomitant with, and easily to be detected at, the period of excitement; (3) those in which the constitutional state is one of stupor and indifference almost verging on coma. As examples of the type, the following cases are cited. A child of two years was suddenly taken ill with fever, intolerance of light, great restlessness, screaming, and twitching of the face and upper extremities. The fever was 104° F. On the fifth day signs of consolidation appeared at the apex of one lung, and on the same day there occurred a crisis, with remission of the cerebral symptoms. The cough became less irritating, and gradually resolved itself into well-defined whooping-cough, this disease having been complicated, no doubt, at its onset by lobar pneumonia. Another case was that of a child of ten years who was seized with an illness in which nervous symptoms were very prominent, and who yielded no physical signs of the pneumonia upon which the condition depended until after the crisis had taken place. The variety which is heralded by coma is instanced by the following case. A young girl was admitted to hospital as suffering from intra-cranial mischief. She was semi-conscious and difficult to rouse, but her quick breathing and the high degree of fever manifested by her—namely, 105° F. —drew attention to the chest, and resulted in the discovery of a patch of pneumonic consolidation at the apex of one lung.

The second variety of pneumonia which is dealt with by the author is that associated with more or less profuse hæmoptysis. This, in Dr. Hood's estimation, is usually found to be associated with apical lesions. These cases are to be distinguished by the following symptoms. The patient, while in the enjoyment of good health, is taken ill with a feverish malady: within a few hours he spits up blood. The hæmoptysis may be large or small, and usually continues for four or six days, during which time the temperature is raised, but rarely, if ever, to the extent met with in the case of ordinary lobar pneumonia. The physical signs are usually apical, and disappear completely. For example, a man of thirty-five years had been feeling unwell for a few days, but not sufficiently to prevent his attending to his business. One morning, when on the point of starting to his work, he was seized with a hæmoptysis. When seen shortly afterwards he was lying on his bed with, beside him, a basin containing about half a pint of blood which he had expectorated with cough. His temperature was 101° F., and he developed some signs of consolidation at the apex of one lung. The sputa became gradually less blood-stained, and the patient made a complete recovery after an illness of seven days' duration. Dr. Hood has seen a number of similar cases, in all of which the lesion was apical. This naturally leads to a suspicion of a tuberculous basis for such cases. The author deliberately concludes that hæmoptysis, when associated with fever and signs of pneumonic consolidation, does not wear the same ominous significance as hæmoptysis generally. A number of examples are quoted to support this thesis. For instance, a man of thirty-five years, while at work, spat up a large quantity of blood-stained sputa. He was a well-nourished and apparently healthy man. The hæmoptysis continued for forty-eight hours, the blood being intimately mixed with the sputa. His temperature when first taken was 101.6° F. By the sixth day the fever had disappeared, and before the conclusion of the patient's stay in hospital—namely, nineteen days—all the signs of consolidation in the right upper lobe which had marked his illness had entirely disappeared. Dr. Hood's contention loses much weight from the circumstance that in one case only of all those upon which he relies for proof do the sputa seem to have been examined for tubercle bacilli.

[1] Clin. Jour., April 11, 1906.

